# Penicillin Allergy, Really?—A Cross-Sectional Mixed-Methods Study in Baden-Württemberg, Germany, to Explore General Practitioner Perspectives on Delabeling Potential in Primary Care

**DOI:** 10.3390/antibiotics15040399

**Published:** 2026-04-15

**Authors:** Regina Poß-Doering, Nicola A. Litke, Elham Khatamzas, Attila Altiner

**Affiliations:** 1 Department of Primary Care and Health Services Research, University Hospital Heidelberg, Medical Faculty, Heidelberg University, 69120 Heidelberg, Germany; nicola.litke@med.uni-heidelberg.de (N.A.L.); attila.altiner@med.uni-heidelberg.de (A.A.); 2 Infectious Disease and Tropical Medicine, Heidelberg University Hospital, 69120 Heidelberg, Germany; elham.khatamzas@med.uni-heidelberg.de

**Keywords:** drug allergies, penicillins, general practice, antibiotic stewardship

## Abstract

**Background:** Most penicillin allergy labels are documented in early childhood and result from events of low risk for allergy. In Germany, evidence-based strategies to evaluate the likelihood of a true penicillin allergy are still lacking. As general practitioner input is indispensable regarding required resources for the implementation of successful delabeling strategies in outpatient care, a mixed-methods study in Baden-Württemberg, Germany explored untapped delabeling potential and conditions for successful initiatives based on their experiences, to support preservation of penicillin as a treatment option and prevent resistance development. **Methods:** A cross-sectional convergent mixed-methods study was conducted with an online survey and semi-structured interviews. The survey link and invitation to participate in an interview was sent to randomly selected publicly available e-mail addresses. Survey data were analyzed descriptively. Qualitative data were analyzed inductively based on thematic analysis. **Results:** n = 101 survey questionnaires and n = 15 interviews were analyzed regarding relevance, experiences, framework conditions, and potential approaches to delabeling. All participants with limited recollection of the index reaction. Most participants considered delabeling a highly relevant topic in general practice. Delabeling efforts were discouraged by lack of time, expertise, and remuneration, and uncertainty due to missing guidelines. Taking a sufficient medical history and, if necessary, subsequent testing were seen as one approach to delabeling. For a standardized approach in primary care, patient and care provider education, precise guideline recommendations, and delabeling expert teams were suggested. **Conclusions:** The findings mirror aspects already identified in international research. A nationwide survey with general practitioners could confirm that addressing necessary resources and systemic adjustments would support effective penicillin allergy delabeling in outpatient care.

## 1. Introduction

In hospitals, up to 20% of patients report a penicillin allergy [1,2,3], making it the most commonly reported type of allergy [1]. The label “penicillin allergy” (PA) influences future antibiotic prescriptions for these patients in both inpatient and outpatient care and can lead to serious problems due to the often lifelong avoidance of beta-lactam antibiotics and the use of alternative substances. These are often associated with a higher risk of infection with Clostridium difficile or Staphylococcus aureus [1], an increased rate of post-operative wound infections, higher mortality, longer hospital stays, and higher costs [4,5]. Unnecessary use of broad-spectrum antibiotics thus contributes to the development of resistant bacteria [1].

Most patients with a documented PA are de facto not allergic, meaning there is no immediate hypersensitivity and they tolerate penicillin and its derivatives [4,5]. Thus, application of a PA label results from events that are low risk for allergy in the vast majority of cases [6]. Often, the label is received early in life, and even vague references to a childhood rash can lead to this label [6,7]. It was observed that approximately 95% of patients who had reported a PA in the past could be identified as penicillin-tolerant [8] following allergological evaluation. Allergy testing and, if necessary, re-exposure can therefore rule out a PA, benefiting patients and preserving important treatment options. In addition to diagnostics and allergological consultation, further strategies are needed to evaluate the likelihood of a true PA, particularly for use by non-allergologists, including a risk-stratified approach in which patients are divided into different risk groups after detailed medical history taking [9].

To offer clarification to as many patients as possible and remove a documented, but inaccurate PA label from patient records, antimicrobial stewardship-based delabeling programs have become increasingly established mainly in inpatient settings. These programs use standardized risk stratification based on history to identify patients who can directly be delabeled or undergo oral provocation testing. In this way, penicillin intolerance can be quickly and reliably ruled out in more than 30% of all patients, allowing penicillin-based antibiotics to be used if required [5,10]. Just like a rational use of antibiotics is a key element in fighting against antibiotic resistance [11], delabeling is an important antibiotic stewardship measure [12]. However, there are still no uniform and broadly applicable recommendations regarding the practical approach [13].

Internationally applied delabeling strategies encompass comprehensive specialist work-up, ranging from serological and skin testing and graded drug challenges, to risk-adapted approaches [14,15,16]. In 2023, the PANDA network (Penicillin Allergy Network Germany) was founded with the aim of improving PA delabeling practices in Germany. The results of a survey the network conducted among German healthcare professionals in 2023 show that a lack of clear recommendations, insufficient financial resources, and unclear defined professional responsibilities are among the barriers to structured PA delabeling [17].

Outpatient allergological testing to facilitate PA delabeling is considered to be costly, not reimbursed at a level that covers costs in the German healthcare system, and therefore usually does not take place [18]. This leads to the assumption that there may be a high number of patients who wrongfully carry the PA label and never received clarification. General practitioners are often the primary care providers for these patients and can assess which resources are required in primary care to implement successful delabeling strategies. It is therefore necessary to gain insights into the untapped potential for delabeling in outpatient care and to explore conditions for successful measures based on the experiences of general practitioners as they are familiar with their own, patient-related, and systemic barriers to delabeling.

Since, to our knowledge, there is still a gap regarding respective insights, this study explored the barriers to delabeling penicillin allergies as perceived by primary care physicians in Baden-Württemberg, Germany, with the aim to derive delabeling concepts and explore which resources and systemic adjustments are perceived as necessary for the implementation of such concepts and develop specific strategies that enable effective delabeling in outpatient care.

## 2. Results

Between February and July 2025, a total of n = 2500 e-mail addresses were contacted one time each in ten mailing waves. N = 122 messages were undeliverable, and n = 270 addresses returned automated responses indicating that they were no longer in use or that messages were no longer being read or answered. The online survey was closed on 31 July 2025, and the response rate was n = 108 (5.1% based on n = 2108), of which n = 101 were fully completed. N = 17 expressions of interest in participating in an interview were received, and n = 15 interviews were conducted with an average duration of 23:18 min. The interview participants were 49.4 years old on average, and 60% were female (n = 9).

Findings derived from both data sources (online survey and interview study) are presented sequentially along the joint themes ‘Relevance and experiences with the penicillin allergy label,’ ‘Framework conditions for delabeling,’ and ‘Potentially suitable approach to delabeling’. Verbatim quotes were extracted from the original qualitative data, translated into English with due diligence, and are provided with indication of the interview number and the transcript position.

### 2.1. Relevance and Experiences with the Penicillin Allergy Label

Online survey participants were asked how many of their patients reported a PA and how many of them, to their knowledge, had been tested. A total of 75% of respondents (n = 81) estimated that up to 10% of patients reported a penicillin allergy, 14.8% (n = 16) reported up to 20%, and 3.7% (n = 4) estimated up to 30%. With regard to testing, 61.1% (n = 66) stated that no one had been tested, 24.1% (n = 26) stated up to 10%, and 4.6% (n = 5) stated up to 20%. Respondents were also asked whether they were familiar with the term ‘delabeling’ and had experience with anamnestic and allergological clarification and the exclusion of suspected PA. Here, 51.9% (n = 56) stated that they were familiar with the term delabeling, 31.5% (n = 34) answered ‘only approximately’, and 10.2% (n = 11) answered ‘no’. A total of 38% (n = 41) stated having experience with delabeling, while 55.6% (n = 60) answered ‘no’.

In light of the potential for antimicrobial resistance development, most interview participants considered the topic to be highly relevant, particularly with regard to general practice, as they often were the first point of contact for patients. Some considered the relevance in their own practice to be low. Others stated that an unconfirmed PA was more relevant to prescribing antibiotics than it actually should be since clarity could be provided by testing. Most participants described actively asking detailed questions during medical history taking or initial consultation when treating infections to assess the potential presence of a PA, taking into account the severity of the reported reaction as an important parameter. A few also mentioned addressing the difference between allergy and intolerance. One participant mentioned that the public should receive more education about this topic, because ‘often people don’t really know what exactly an allergy or an intolerance is.’ (15, Pos. 49).

Interview participants’ estimates of the proportion of patients who reported a PA ranged from 5 to 20%, and the estimate of actual PA ranged from 1 to 5%, as just few immediate reactions and mostly intolerance appeared to be described. It was also noted that younger people more often reported a PA than older patients. One difficulty mentioned was that the exact extent of the reaction was often unknown, as it had occurred long ago and allergy records were generally only available in a few cases.

‘Many people write this down in the medical history form or mention it, so I usually ask them if they know the exact name of the medication and what symptoms they experienced. Most of them don’t know. Most of them can’t say much about it.’

(04, Pos. 4)

### 2.2. Framework Conditions for Delabeling

**Figure 1:**
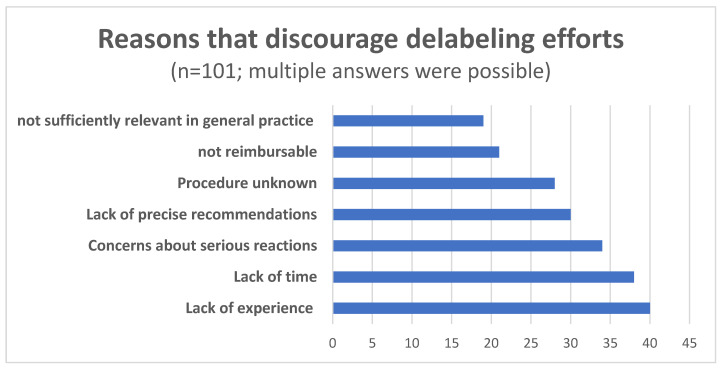
Distribution of the most frequently cited reasons that discourage delabeling.

Survey participants could indicate reasons that discouraged them from delabeling. Multiple answers were possible regarding effort, knowledge, personal responsibility, availability of precise recommendations, concerns or legal consequences, experience, time, risk assessment, assessment of alternative antibiotics, and billing. One participant indicated that benefits did not outweigh the risk of testing, and n = 8 responses were attributed to ‘Not within my responsibility’, n = 9 to the availability of sufficient alternative antibiotics, and n = 18 to ‘Fear of legal consequences’. Under ‘Other reasons’, n = 3 respondents noted that patients were often strongly convinced that they had a PA and were not willing to be tested. Figure 1 describes the most frequently selected reasons.

In the interviews, all participants reported that patients rarely asked to what extent it would be possible to evaluate whether they actually had a PA, due to concerns about potential reactions. Several participants shared their concerns and reflected on them. In general, appropriate training of the practice team and emergency equipment in the practice were seen as necessary in order to be able to perform any form of delabeling process in general medical practices. In addition to the factors of time, expertise, and remuneration, uncertainty due to a lack of guidelines and binding recommendations for delabeling and uncertainty about the validity of available tests were also discussed. It was expressed that ‘it would be good to have something in hand to say, ‘You don’t have to go to an allergist first, we can just go through these questions here and possibly also do an exposure test.’ (09, Pos. 26) to start a delabeling process.

Most participants stated that they did not see themselves and their teams as responsible or sufficiently qualified for delabeling efforts and would rather send patients to specialized practices or teams. As a result, they felt it was more adequate to refer patients to allergists, dermatologists, or a university hospital for testing.

‘I wouldn’t do that in my practice because we’re not equipped to deal with anaphylaxis. And we don’t want to have an incident in the practice. I’m very happy when things are running smoothly, and I would always refer such cases to immunologists or dermatologists who perform allergy testing. They are equipped for it, they have everything ready to go, their emergency kit, their laboratories, or their training for the assistant, we don’t have any of that.’

(02, Pos. 36)

### 2.3. Potentially Suitable Approach to Delabeling

**Figure 2:**
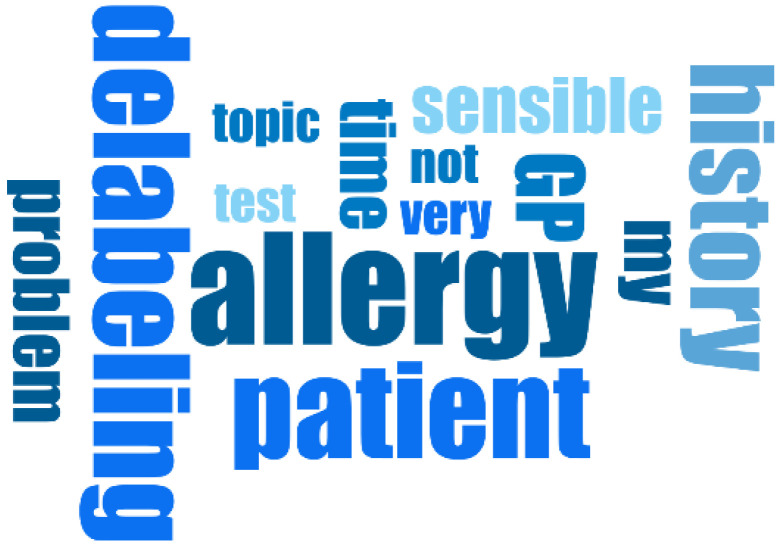
The most commonly used words in the free text comments.

Potentially suitable approaches to delabeling were outlined in n = 27 comments in the free text field of the online survey. It was pointed out several times that a PA could often be ruled out by taking a sufficient medical history and, if necessary, subsequent dermatological or allergological testing. Several respondents noted that an intensified approach to delabeling was not possible in general medical practices because it involved a lot of effort, was associated with risks, and was not reimbursed. It was also suggested to set up specialized centers, either regionally or affiliated with university hospitals, which could routinely carry out tests with appropriate specialist staff, also because it often would be difficult to refer patients to outpatient specialists. In addition, development of specific delabeling recommendations for the outpatient setting was proposed. Most commonly used words in the comments are shown in Figure 2:

The interview participants stated that a standardized approach to delabeling should also be possible in general medical practices. Suggestions on how this could be supported included aspects of educating and informing those affected, training and continuing education for care providers, binding and precise recommendations, development of guidelines, discussion and exchange in quality circles, and special contact points with appropriately qualified personnel. It was also mentioned that professional associations, journals, associations of statutory health insurance physicians, and the Federal Institute for Public Health could contribute to raising awareness of the issue and promoting awareness of the relevance of delabeling.

‘... it would be good to know that penicillin is an option and that the patient then thinks, yes, maybe I’ll try that again. I think I would then be more willing to give it another go.’

(12, Pos. 12)

‘Of course, it’s a sensible component, as it also raises awareness again, but I would find it fundamentally beneficial in the context of continuing education, antibiotics, rational use, and such, where resistance is also an issue, that the topic of penicillin allergy is definitely included in this context.’

(11, Pos. 57)

## 3. Discussion

Findings of this mixed-methods study confirm that substantial barriers to PA delabeling exist in German primary care that need to be addressed to facilitate broader implementation. The majority of participants in the survey and the qualitative study considered PA delabeling a highly relevant topic for general practice. Half of the survey participants indicated being familiar with the term ‘delabeling’, and less than 40% claimed to have some experience with it. The most prominently indicated reasons for not pursuing delabeling efforts in both data sources were a lack of experience with adequate processes, a lack of time during daily practice, concerns about serious allergic reactions, and a lack of national guidelines and specialized points of contact with expert teams. In addition, participants reported that patients often had limited recollection of the index reaction, and rarely asked for testing to confirm or exclude a PA. Intensified approaches to delabeling in the outpatient setting were reflected regarding effort, risks, and reimbursement. Educating and informing patients, training and continuing education for care providers, and precise recommendations and guidelines, as well as peer exchange in quality circles were seen as facilitators and deemed necessary to increase and promote delabeling efforts.

For Europe, PA delabeling studies are still limited and mostly focus on small-cohort implementation, for instance, in Norway [19], France [20], the Netherlands [21], and UK [22]. Since studies mainly focused on clinical aspects and clinicians’ determinants, there is still a need for a deeper understanding of patient factors that determine acceptance and adherence and might restrict effectiveness of delabeling strategies [6]. A recent study in the UK hospital setting explored attitudes and acceptability of patients and healthcare professionals regarding a delabeling strategy for identified low-risk patients. Findings indicated high levels of acceptability particularly among patients, and issues of knowledge, risk, governance and workforce were identified as key determinants to be considered in future strategy planning. It was concluded that the findings underscored key factors such as required infrastructure for delabeling interventions, trained staff, suitable locations, referral pathways, and expertise among care providers [23]. For Germany, a nation-wide survey generated insights into attitudes and barriers to delabeling efforts from the perspectives of healthcare professionals and concluded that PA delabeling is not yet routinely integrated in clinical pathways due to missing guidelines, limited resources and unclear role definitions. It was also found that the strong potential for the implementation of structured delabeling strategies requires support through adequate training, clear protocols and sufficiently resourced antimicrobial stewardship teams [17]. The factors and aspects identified in both these studies were also reflected on by participants in this present study and they clearly stated the relevance of delabeling regarding the preservation of penicillins as a treatment option and prevention of resistance development.

As already has been suggested [24] and in light of the high potential and rates of safe delabeling, adequate guideline recommendations are desirable. This also applies to educational responses to patient and practitioner concerns and risk perception, as well as expert guidance, as these appear to be beneficial to a stronger uptake of delabeling efforts, particularly in primary care.

PA delabeling has been shown to be safe and effective, even when undertaken by non-allergy specialists [23]. In this study, a major argument general practitioners refrained from prescribing penicillin was the lack of reliable patient information with details of the index reaction. Most PA diagnoses originate in childhood and are associated with non-allergic events, low-risk reactions, or true allergies that may have waned over time [6]. While this may explain fragmented patient reports, participants also perceived patient hesitancy towards evaluation of suspected PA and felt this reluctance should be respected. They suggested awareness campaigns and educational initiatives to increase patient health literacy and risk perception regarding PA.

Participants in this study indicated that their history taking included asking about drug allergies, yet most of them did not pursue testing with regard to a suspected PA. However, allergic testing in cases of PA based on medical history is a routine medical procedure and oral or parenteral exposure to the suspected drug is considered the gold standard for ruling out the drug allergy [4]. Yet, the participants voiced their concern about potentially serious allergic reactions caused by exposure to penicillin and cautioned about taking such a risk in primary care settings. Mediating measures such as emergency equipment, binding guideline recommendations, and expert support were deemed indispensable. Besides patient awareness and education, future delabeling initiatives should therefore consider educational measures for primary care practice teams to address these concerns and support adequate risk assessment and reimbursement.

A patient’s allergy history often guides the choice of prescribed antibiotics [5]. Penicillin and other β-lactam antibiotics are considered first-line therapies for a number of bacterial infections since they are effective, safe, and narrow antimicrobial spectrum. Once a patient is labeled with a PA, prescribing penicillin or β-lactams often is avoided out of concern for a potential cross-reactivity [25]. However, unnecessarily restricting antibiotic therapy options increases harm associated with second-choice therapy options for patients and leads to increased use of broad-spectrum antibiotics, higher rates of Clostridium difficile infections or colonization [26], less oral antibiotic treatment options and more hospitalizations [27], poorer outcomes, higher costs, and spread of antimicrobial resistance [26]. On the other side, PA delabeling is considered to be an effective and safe antimicrobial stewardship intervention that improves access to and use of first-line therapy, reduces broad-spectrum antibiotic use, and saves costs [5,28]. The evidence-based guideline for suspected antibiotic allergy developed in the Netherlands concludes that applying proven PA delabeling measures, such as structured history taking, risk stratification, skin testing and/or direct oral challenge, can lead to safe use of β-lactams for approximately 90% of patients with an assumed PA. The guideline provides recommendations for allergy characterization and patient eligibility for PA delabeling and emphasizes that avoiding certain antibiotics in case of suspected true and severe antibiotic allergy should be strongly advised [29].

### Strengths and Limitations

Studies exploring care provider perspectives on PA delabeling are scarce for Germany. To the best of our knowledge, this study is the first one to integrate quantitative and qualitative data to provide a base for future recommendations for safe delabeling in primary care. The relevance of the topic is underlined by the strong interest of general practitioners in participating in this study. The sample included male and female physicians of different age and professional experience. Analysis of the qualitative data facilitated identification of relevant key and sub-themes across all interviews in a systematic yet flexible approach. Reporting is based on scientifically valid criteria [30]. The individual telephone interviews facilitated comprehensive insights into personal perceptions and experiences, and accommodated for the participants’ time resources.

A limitation of this study is the focus on general practitioner perspectives in one federal state only as it remains unclear whether general practitioners in other federal states could have provided different perspectives based on regionally relevant aspects. General practitioners who abstained from participating or who were not included in the random sample might have contributed different perceptions; thus, selection bias at different levels and a potential lack of representativity might be present regarding the survey sample size. The patient perspective was not investigated. It is possible that the invitation to the survey did not reach individual physicians in a number of cases as it was not possible to determine whether team or individual e-mail addresses were retrieved and used. Participating general practitioners were possibly particularly interested in PA delabeling and had a positive attitude towards the research project. Though difficult to assess, selection bias and social desirability regarding chosen responses in the interviews and the survey cannot be excluded and may have led to overestimating knowledge about delabeling. However, to prevent socially desirable responses, dichotomous response options were not used in the survey. There is also a risk of interviewer bias. To counteract possible bias in the reported findings, the research team discussed and consented individual work steps and data analysis.

## 4. Materials and Methods

### 4.1. Study Design and Recruitment

A cross-sectional convergent mixed-methods design [31] was chosen with an anonymous online survey (five questions, one free text field) and guided semi-structured interviews with general practitioners practicing in the federal state of Baden-Württemberg, the third largest state in Germany. Recruitment was carried out by sending brief information about the study and a link to the survey to approximately half of the publicly available e-mail addresses of general practices in Baden-Württemberg. E-mail addresses were retrieved by an online search and sorted in alphabetical order. The e-mail was sent to a random sub-set of addresses across Baden-Württemberg and all letters of the alphabet. Based on experiences from other studies, a response rate of 3% was expected for this explorative study. PA delabeling was mentioned in the study title, but no further explanation or definition was provided. At the end of the survey, participants were informed about the optional interview and a contact e-mail address was provided. Physicians could participate in the online survey, a telephone interview, or both.

Potential interview participants were contacted by phone or e-mail after expressing interest by e-mail, provided with detailed information about the study, and sent the written study information, a consent form, and a short socio-demographic questionnaire. All interested physicians were contacted by phone or via e-mail to inform them about the aim of the study and to agree on a date and time for the interview by the study team. An expense allowance was offered for participation in the interview.

### 4.2. Data Collection

After conducting a literature search, the study team compiled a list of questions focusing on aspects to be identified. Following prioritization by relevance, an online questionnaire and an interview guide were developed focusing on the topics of ‘relevance and experiences with penicillin allergy’, ‘framework conditions for a re-evaluation’, and ‘potentially suitable approach’ (see Supplementary Materials File S1). In an iterative process, the survey and socio-demographic questionnaires and the interview guide were discussed, adapted and consented by the research team during several feedback loops. The survey was made available on a secure online platform hosted by the University Heidelberg, Germany. Telephone interviews were conducted by an experienced researcher (RPD) after written consent was obtained. Socio-demographic characteristics were collected paper-based and only from interview participants. The aim was to conduct 8–15 interviews in order to classify identified aspects and recommendations as relevant with sufficient certainty and achieve thematic saturation [32]. No survey sample size calculation was performed.

### 4.3. Data Analysis

Survey data were imported from the online platform into IBM SPSS Statistics (IBM, Armonk, New York, USA, version 29.0.0.0) for descriptive analysis. Socio-demographic data were organized in Microsoft Excel. All quantitative data were visualized in Microsoft Excel. All qualitative data were audio-recorded, pseudonymized, and transcribed verbatim using the artificial intelligence software noScribe (Kai Dröge, Frankfurt/Main, Germany, version 0.3) [33]. All transcripts were meticulously checked for plausibility and amended where necessary by study team members and then analyzed inductively in MAXQDA (Verbi Software, Berlin, Germany, version 22.7.0) based on thematic analysis [12,13]. Transcripts were not checked or corrected by participants. Coding was carried out by two experienced researchers (RPD, NL) and consented in a series of meetings. Quantitative and qualitative data were first analyzed separately and subsequently integrated to identify common concepts across both data sources. All collected data were organized and stored on secure servers at the University Hospital Heidelberg.

## 5. Conclusions

This study set out to explore general practitioner perspectives regarding barriers to PA delabeling to provide an initial base for the development of specific future concepts. The resources and systemic adjustments that the participants perceived as necessary for the implementation of such concepts mirror aspects already identified in international research and guidelines. Conducting a nationwide survey with general practitioners in Germany could be a next step to confirm that addressing these issues would support effective PA delabeling in outpatient care and create an appropriate delabeling strategy for primary care.

## Data Availability

All data generated and analyzed within this study are stored on a secure server at the Department of Primary Care and Health Services Research, University Hospital Heidelberg, Germany. De-identified sets of these data can be made available by the corresponding author on reasonable request.

## Supplementary Materials

The following supporting information can be downloaded at https://www.mdpi.com/article/10.3390/antibiotics15040399/s1, File S1: Survey and interview guide topics.

## Abbreviations

The following abbreviations are used in this manuscript:

PA	Penicillin allergy
PANDA	Penicillin Allergy Network Germany
