# Supplementary material for: Penicillin Allergy, Really?—A Cross-Sectional Mixed-Methods Study in Baden-Württemberg, Germany, to Explore General Practitioner Perspectives on Delabeling Potential in Primary Care
Source: Antibiotics (Basel). 2026 Apr 15;15(4):399. doi: 10.3390/antibiotics15040399 (PMC13114247; doi:10.3390/antibiotics15040399)
Supplement: Supplementary file 1 [file antibiotics-15-00399-s001.zip › antibiotics-4228294-supplementary.pdf]

**Supplementary Materials**

## File S1: Survey and interview guide topics

### Survey questions:

1. From your perspective, how many patients in your practice report a penicillin allergy? (indicate a percentage)
2. To the best of your knowledge, how many of these patients underwent testing? (indicate a percentage)
3. Do you have any idea what the term “delabeling” of a penicillin allergy might mean? (Yes/No)
4. Do you personally have any experience with penicillin allergy delabeling? (Yes/No)

The label „penicillin allergy“ can have a number of negative consequences. “Delabeling” involves a medical and allergy evaluation and the exclusion of the suspected diagnosis. Do you think

It is an important task. (Yes/No)

Delabeling is not sufficiently relevant in general practices with regard to the effort. (Yes/No)

5. What factors are currently preventing you from delabeling? (Multiple answers allowed)
   Don’t know the process yet.
   Not my responsibility
   Lack of precise recommendations
   I am concerned about serious allergic reactions
   I am concerned about legal consequences.
   I don’t have any experience.
   Lack of time
   From my point of view, advantages do not outweigh the risk of a serious allergic reaction.
   I think there are sufficient altenative antibiotics
   I cannot be reimbursed for delabeling.
   None, I conduct delabeling.
   Other

Free text: Anything else you'd like to tell us?

**Interview guide**

1) How would you assess the relevance of this topic to primary care?

   Please describe your experience with patients who have a confirmed penicillin allergy. How do you determine that a patient has a penicillin allergy? How do you confirm the presence of an allergy? Do you talk about the difference between allergy and intolerance?

2) What does an allergy mean for the care of these patients?

   How do you approach treatment planning when antibiotics are necessary? What needs to be considered for long-term care (5–10 years)?

3) To what extent is re-evaluation/testing taken into account in treatment planning?

   For which patient groups might this be of interest? How many patients come to you and ask for a re-evaluation? (active outreach, motivation) What is the patient perspective on delabeling?

4) What discourages you or your colleagues from conducting a re-evaluation for all individuals with a suspected or diagnosed penicillin allergy? (Reasons)

   What type of testing would you personally perform? What would need to change for re-evaluation to be performed as standard practice? In this context, what are your thoughts on SOPs/guidelines/training/reimbursement?

5) In your opinion, what delabeling procedure would be suitable for general practice settings?

   How is this handled in your practice (when there are multiple providers)?

   What has been your experience with conducting provocation tests? What is the evidence regarding such tests? What overall conditions would be necessary?

6) Closing remarks: What aspects have we overlooked?
